# Prognostic value of RKIP and p-ERK in gastric cancer

**DOI:** 10.1186/1756-9966-31-30

**Published:** 2012-03-31

**Authors:** Yoshitaka Fujimori, Mikito Inokuchi, Yoko Takagi, Keiji Kato, Kazuyuki Kojima, Kenichi Sugihara

**Affiliations:** 1 Department of Surgical Oncology, Tokyo Medical and Dental University, 1-5-45, Yushima, Bunkyo-ku, Tokyo 113-8519, Japan; 2 Department of Translational Oncology, Tokyo Medical and Dental University, 1-5-45, Yushima, Bunkyo-ku, Tokyo 113-8519, Japan; 3 Department of Minimum Invasive Surgery, Tokyo Medical and Dental University, 1-5-45, Yushima, Bunkyo-ku, Tokyo 113-8519, Japan

## Abstract

**Background:**

The mitogen-activated protein kinase (MAPK) signaling pathway participates in several steps of tumour development and is considered a prominent therapeutic target for the design of chemotherapeutic agents. We evaluated the expressions of extracellular signal-regulated kinase (ERK), mitogen-activated protein kinase (MEK), an upstream regulator of ERK, and Raf kinase inhibitor protein (RKIP), and investigated correlations of these expressions with clinicopathological features and outcomes in gastric cancer.

**Methods:**

Tumour samples were obtained from 105 patients with gastric adenocarcinomas who underwent radical gastrectomy. The expressions of phosphorylated ERK (p-ERK), phosphorylated MEK (p-MEK), and RKIP were analysed by immunohistochemical staining.

**Results:**

Expression of RKIP, p-MEK, and p-ERK was found in 69 (66%), 54 (51%), and 64 (61%) of all tumours, respectively. RKIP expression negatively correlated with the depth of invasion (p < 0.001), lymph node involvement (p = 0.028), and Union for International Cancer Control (UICC) stage (p = 0.007). RKIP expression was associated with significantly longer relapse-free survival (RFS) (p = 0.0033), whereas p-MEK was not (p = 0.79). Patients with p-ERK expression had slightly, but not significantly shorter RFS than those without such expression (p = 0.054). Patients with positive p-ERK and negative RKIP expression had significantly shorter RFS than the other patients (p < 0.001). The combination of RKIP and p-ERK expression was an independent prognostic factor (hazard ratio, 2.4; 95% confidence interval, 1.3 - 4.6; p = 0.008).

**Conclusions:**

Our results demonstrated that loss of RKIP was associated with tumour progression and poor survival. Negative RKIP expression combined with positive p-ERK expression was an independent predictor of poor outcomes in patients with gastric cancer.

## Background

Gastric cancer remains the second most common cause of cancer-related death worldwide [1,2]. Many Asian countries, including China, Japan, and Korea, still have very high incidences of and mortality from gastric cancer. Despite progress in early diagnosis of gastric cancer, many patients present with unresectable, locally advanced, or metastatic disease associated with an extremely poor prognosis. Most cases of advanced gastric cancer remain incurable, with a median survival of only 6-12 months even in patients who receive intensive chemotherapy [3-7]. Trastuzumab, a monoclonal antibody against human epidermal growth factor receptor 2 (HER2), is therapeutically effective in gastric cancer. However, 22% of all advanced or metastatic gastric cancers showed HER2 overexpression in one clinical trial [8]. A better understanding of the etiologic factors and molecular mechanisms underlying the pathogenesis of gastric cancer is thus essential for improved outcomes.

Mitogen-activated protein kinases (MAPKs) are serine/threonine kinases that are activated in response to a variety of external signals. Extracellular signal-regulated kinases (ERK) comprise one subclass of MAPKs that can be activated by various receptor tyrosine kinases, cytokine receptors, G proteins, and oncogene products through phosphorylation by MAPKs or ERK-activated protein kinase (MEK). On activation of the MAPK cascade, ERK is phosphorylated by MEK on threonine and tyrosine residues and translocates from the cytoplasm to nucleus, where ERK phosphorylates several nuclear targets, including transcription factors [9]. After stimulation, ERK is phosphorylated by MEK, from which it then dissociates. The MEK-mediated phosphorylation of ERK, especially tyrosine phosphorylation, is prerequisite for the dissociation of ERK from MEK. Dissociated ERK then enters the nucleus by either passive diffusion or active transport mechanisms [9]. ERK is implicated in various cellular processes, including proliferation, differentiation, apoptosis, and transformation.

Raf kinase inhibitor protein (RKIP), also termed phosphatidylethanolamine binding protein (PEBP)-1, is a 20-25 kDa globular protein that belongs to the PEBP family, encompassing more than 400 members [10]. RKIP is supposed to bind to Raf-1 and inhibit Raf-1-mediated phosphorylation of MEK [11,12]. As a modulator of signaling pathways, RKIP also affects various cellular processes [13]. Deviant control of the MAPK cascade has been implicated in the development of human neurodegenerative diseases, such as Alzheimer's disease, Parkinson's disease, and amyotrophic lateral sclerosis, as well as various types of human cancer. Many Ras and B-Raf mutations occur in human cancer [14].

The purpose of this study was to investigate the expression of phosphorylated ERK (p-ERK) and its upstream regulating signals such as phosphorylated MEK (p-MEK) and RKIP in human gastric cancer and to evaluate relations of the expressions of these proteins to clinicopathological variables and outcomes.

## Methods

### Patients

February 2004 through December 2007 we studied 105 patients who underwent curative gastrectomy (R0) for primary gastric adenocarcinomas penetrating beyond the muscularis mucosa at the Department of Esophagogastric Surgery, Tokyo Medical and Dental University. This study was conducted due to Declaration of Helsinki [15], and approved by Institutional Review Board of the Tokyo Medical and Dental university. Each tumour was classified according to the tumour-node-metastasis (TNM) classification recommended by the Union for International Cancer Control (UICC). All patients were evaluated for recurrent disease by examinations of tumour markers or by diagnostic imaging, including computed tomography, ultrasonography, magnetic resonance imaging, and endoscopy, every 3-6 months. No patient received neoadjuvant therapy. The median follow-up time was 55 months (range, 37-84). Recurrent disease was diagnosed in 45 patients (43%) and was the cause of death in 40 (38%) patients.

### Immunostaining of p-MEK and p-ERK and RKIP

Immunohistochemical staining was carried out by the streptavitin-biotin method using a Histofine SAB-PO kit (Nichirei Co., Tokyo, Japan). Polyclonal rabbit antibody against p-ERK was purchased from Abcam^® ^(Cambridge, UK), monoclonal Rabbit antibody against p-MEK 1/2 (Ser221) was purchased from Cell Signaling Technology, Inc. (Beverly, MA, USA), and RKIP antibody was purchased from Santa Cruz Biotechnology, Inc. (Santa Cruz, CA, USA). All available haematoxylin-and-eosin-stained slides of the surgical specimens were reviewed. For each case, representative paraffin blocks were selected for immunohistochemical studies. Three-micrometer-thick sections were cut from each formalin-fixed, paraffin-embedded tissue block. After deparaffinisation and rehydration, antigen retrieval treatment was carried out at 98°C (microwave) for 15 min in 10 mmolL sodium citrate buffer (pH 6.0), followed by treatment with 3% hydrogen peroxide for 15 min to quench endogenous peroxidase activity. Nonspecific binding was blocked by treating the slides with 5% EzBlock (including 10% normal goat serum) for 10 min at room temperature. The slides were incubated with primary antibodies including p-ERK (dilution 1:50), p-MEK (1:50), and RKIP (1:100) overnight at 4°C. Immunodetection was performed by the conventional streptavidin-biotin method with peroxidase-labeled Nichirei SAB-PO kits. Diaminobenzidine substrate was used for colour development. The slides were counterstained with 1% Mayer's haematoxylin. Expression levels of p-ERK, p-MEK, and RKIP were classified into groups based on staining intensity and positive frequency. We counted stained cells under a microscope to derive the scores. The cytoplasmic and nuclear staining patterns were separately quantified, using a semiquantitative system to evaluate and grade the immunostaining pattern, as successfully applied by others [16]. Staining intensity was scored into four grades: 0 (none), 1 (weak positive), 2 (moderate positive), and 3 (strong positive). Staining extent (positive frequency) was also scored into four grades: 0 for complete absence of staining, 1 for < 10%, 2 for 10% to 50%, and 3 for tumours with staining of 50% or more cells. Composite scores were derived by multiplying the intensity score by the staining extent score. For statistical analysis, composite scores of ≥4 were defined as cytoplasmic expression positive, and scores of < 4 were considered negative. We assessed the cytoplasmic expressions of RKIP and MEK and the nuclear expression of ERK as described previously [16,17].

### Statistical analysis

The χ^2 ^test was used to test possible associations between the expression of p-ERK, p-MEK, or RKIP and clinicopathological factors. It was also used to assess correlations between p-ERK, p-MEK, and RKIP expressions. Kaplan-Meier curves were plotted to assess the relations of p-ERK, p-MEK, and RKIP expressions to relapse-free survival (RFS). Survival curves were compared using the log-rank-test. P-values of less than 0.05 (P < 0.05) were considered to indicate statistical significance. Multivariate Cox proportional-hazards regression models were used to assess the prognostic significance of p-ERK, p-MEK, and RKIP expressions and of several clinicopathological factors. Statistical analysis was carried out with the use of SPSS Base, version 17.0 and SPSS Advanced models, version 17.0 (SPSS Inc., Chicago, IL, USA) software.

## Results

**Figure 1:**
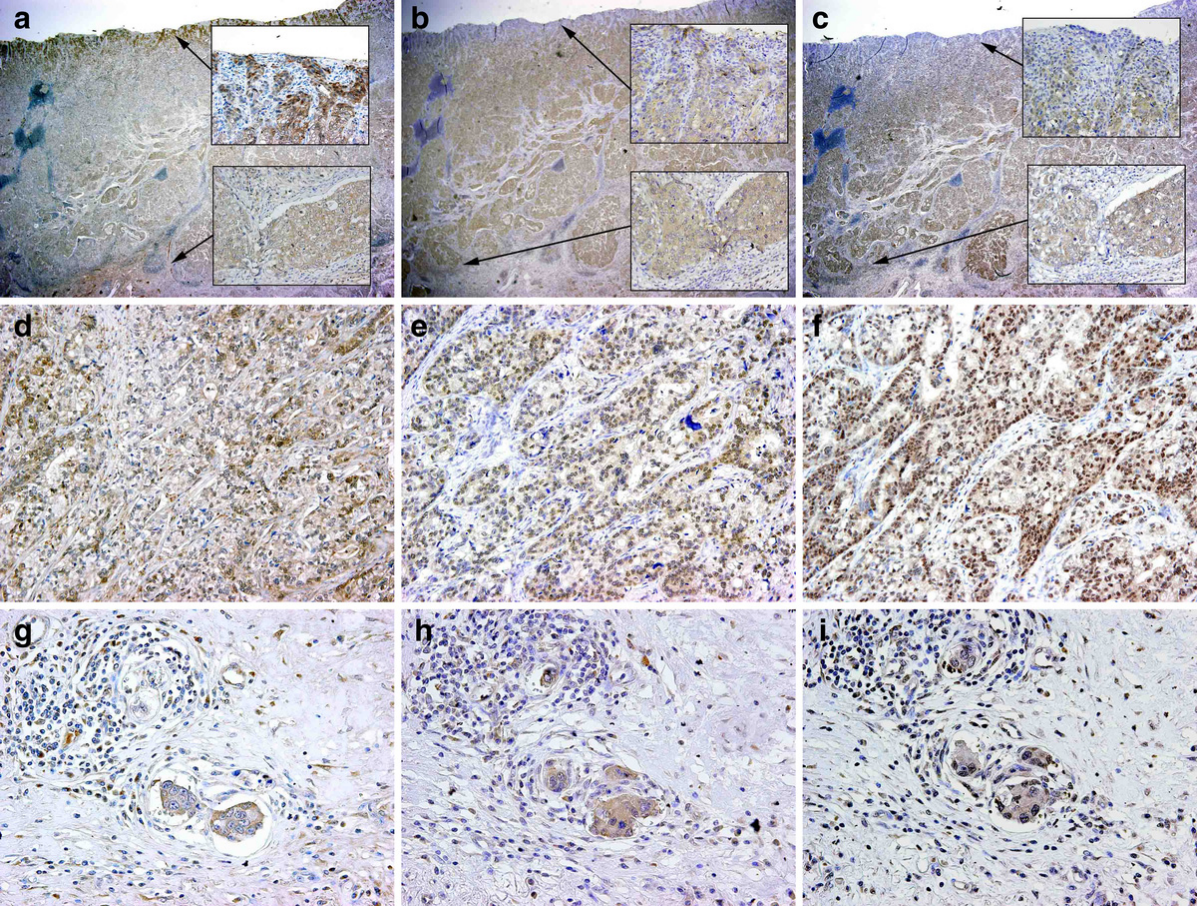
**Representative gastric carcinomas showing immunostaining for RKIP predominantly in the cytoplasm, (a), immunostaining for p-MEK predominantly in the cytoplasm (b), and immunostaining for p-ERK in the nucleus and the cytoplasm (c); magnification, 2×**. The upper inset shows a surface site of tumour and the lower inset shows a site of deep invasion (a - c); magnification, 40×. Metastatic lymph nodes showing immunostaining for RKIP in the cytoplasm (d), for p-MEK in the nucleus (e), and for p-ERK with strong intensity in the nucleus (f); magnification, 40×. Tumour cells associated with venous invasion showing immunostaining for RKIP with weak intensity (g), for p-MEK (h), and for p-ERK in the nucleus (i); magnification, 40×.

RKIP, p-MEK, and p-ERK were respectively expressed by 69 (66%), 54 (51%), and 64 (61%) of all tumours (Figure 1a-c). RKIP expression was mainly observed in the cytoplasm of tumour or non-tumour cells. Expressions of p-MEK and p-ERK were found in both the cytoplasm and nucleus. Expressions of RKIP, p-MEK, and p-ERK were respectively detected in 5 (19%), 9 (35%), and 21 (81%) of 26 metastatic lymph nodes obtained from patients with recurrent disease (Figure 1d-f). Expression of p-ERK was found mainly in the nuclei of metastatic tumour cells. These proteins were also detected in tumour cells associated with venous invasion (Figure 1g-i). No p-ERK or p-MEK staining was detected in normal gastric mucosa. The expression of p-MEK positively correlated with the expressions of RKIP (p = 0.042) and p-ERK (p = 0.007), whereas there was no relation between RKIP and p-ERK expressions (p = 0.98) (Table 1). RKIP expression negatively correlated with the depth of invasion (p < 0.001), lymph node involvement (p = 0.028), and UICC stage (p = 0.007). RKIP was more commonly found in differentiated type than in undifferentiated type tumours (p = 0.042). The expressions of p-ERK and p-MEK significantly correlated with gender (p = 0.027, p = 0.036, respectively), but were not related to any other clinicopathological factor (Table 2).

**Table 1:** Correlations among RKIP, p-MEK, and p-ERK expressions

	p-MEK			p-ERK		
	negative	positive	p	negative	positive	p
RKIP						
negative	25	16	0.042	14	27	0.98
positive	26	38		22	41	
p-MEK						
negative				24	27	0.007
positive				12	42	

**Table 2:** Clinicopathological factors and expression of RKIP, p-MEK, and p-ERK

		cytoplasmic			cytoplasmic			nuclear		
		RKIP			p-MEK			p-ERK		
	n	negative	positive	p	negative	positive	p	negative	positive	p
Age										
> 70	46	16	30	0.43	20	26	0.36	17	29	0.61
≦ 70	59	25	34		31	28		19	40	
Gender										
female	21	6	15	0.40	15	6	0.036	12	9	0.027
male	84	35	49		36	48		24	60	
Histopathology (WHO)										
pap	12	3	9	0.20	5	7	0.34	5	7	0.99
tub1	15	2	13		5	10		5	10	
tub2	27	11	16		13	14		10	17	
por1	14	7	7		5	9		4	10	
por2/sig	31	15	16		20	11		10	21	
muc	6	3	3		3	3		2	4	
Histopathology (2 groups)										
differentiated	54	16	38	0.042	23	31	0.21	20	34	0.54
undifferentiated	51	25	26		28	23		16	35	
Depth of invasion										
T1b/2	32	4	28	< 0.001	14	18	0.51	12	20	0.65
T3/4	73	37	36		37	36		24	49	
LN metastasis										
negative (N0)	35	8	27	0.028	16	19	0.68	15	20	0.19
positive (N1/2/3)	70	33	37		35	35		21	49	
Distant metastasis or recurrence										
negative	68	19	49	0.002	33	35	0.99	27	41	0.17
positive	37	22	15		18	19		9	28	
Stage										
I/II	53	14	39	0.007	24	29	0.50	19	34	0.73
III/IV	52	27	25		27	25		17	35	

**Figure 2:**
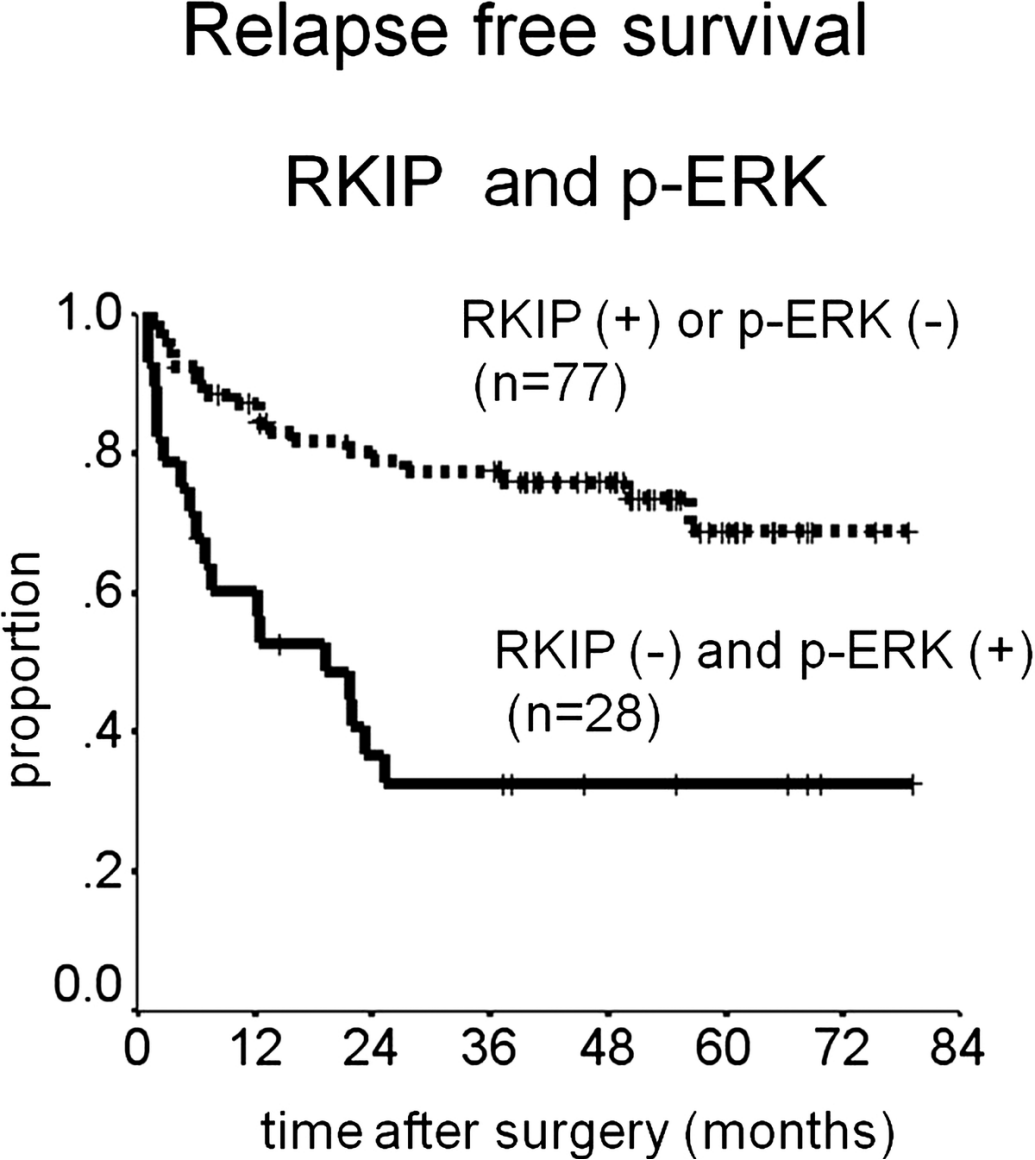
**Kaplan-Meier curves for the relapse-free survival of patients with expression of RKIP and p-ERK**.

RKIP expression was associated with significantly longer RFS (p = 0.003), whereas p-MEK was not (p = 0.79). The presence of p-ERK expression was associated with slightly, but not significantly shorter RFS than the absence of such expression (p = 0.054) (Table 3). Patients with positive p-ERK and negative RKIP expression had significantly shorter RFS than the other patients (p < 0.001) (Figure 2). The prognostic relevance of positive p-ERK expression combined with negative RKIP expression was therefore assessed using a multivariate proportional-hazards model adjusted for established clinical prognostic factors (i.e., age, gender, histopathology, depth of invasion, lymph node involvement). The combination of RKIP and p-ERK expression was found to be an independent prognostic factor (hazard ratio [HR], 2.4; 95% confidence interval [CI], 1.3 - 4.6; p = 0.008). Histopathological type and depth of invasion were also independent prognostic factors (HR, 2.1; 95% CI, 1.0 - 4.2; p = 0.043 and HR, 4.7; 95% CI, 1.0-22; p = 0.048, respectively) (Table 3).

**Table 3:** Prognostic factors in multivariate Cox proportional-hazards regression models for RFS

	Univariate^a)^		Multivariate 1^b)^			Multivariate 2^c)^		
	5-yr RFS^d)^	p	HR	95%CI	p	HR	95% CI	p
Age								
> 70	73							
≦ 70	51	0.094						
Gender								
female	74							
male	56	0.22						
Histopathology								
differentiated	79		1.0			1.0		
undifferentiated	42	0.001	2.2	1.1 - 4.4	0.035	2.1	1.0 - 4.2	0.043
Depth of invasion								
T1/2	93		1.0			1.0		
T3/4	46	0.002	4.8	1.0 - 23	0.048	4.7	1.0 - 22	0.048
Lymph node metastasis								
negative (N0)	83		1.0			1.0		
positive (N1/2/3)	48	0.002	1.6	0.59 - 4.5	0.34	1.6	0.59 - 4.5	0.35
RKIP								
positive	66		1.0					
negative	44	0.003	1.7	0.89 - 3.3	0.11			
pMEK								
negative	62							
positive	55	0.79						
pERK								
negative	75		1.0					
positive	49	0.054	2.0	0.93 - 4.2	0.078			
Combined expression								
RKIP(+) or p-ERK(-)	69					1.0		
RKIP(-) and p-ERK(+)	33	< 0.001				2.4	1.3 - 4.6	0.008

a) log-rank test

b) analysed factors: Histopathology, Depth of invasion, Lymph node metastasis, RKIP, and pERK

c) analysed factors: Histopathology, Depth of invasion, Lymph node metastasis, and Combined expression of RKIP and pERK

d) the rate of 5-year relapse free survival

## Discussion

Our study showed that loss of RKIP expression and overexpression of ERK in the MAPK signaling pathway were associated with survival in patients with invasive gastric cancer. Few previous studies have examined correlations among RKIP, MEK, and ERK expressions in samples of human cancer. RKIP is considered to be a signal transduction modulator and metastasis suppressor that inhibits the upper MAPK signaling pathway. RKIP binds to Raf-1 and prevents MAP kinase signaling in response to growth factors [11,13]. Loss of RKIP is thought to induce activation of MEK and ERK; however, evidence supporting this negative correlation was not found in the present study. RKIP is missing or depleted in a number of metastatic tumours [10], especially human breast [18] and colorectal cancer [19]. In the present study, RKIP expression was lost in many metastatic lymph nodes, consistent with the results of those investigations. In the patients with gastrointestinal stromal tumours (GISTs), RKIP expression levels correlate with clinical-pathological factors, and loss of RKIP expression is associated with poor survival [20]. RKIP expression has been reported to be lower in gastric carcinoma than in normal gastric tissue [21]. Loss of cytoplasmic RKIP was significantly linked to poor survival of patients with gastric cancer [16,22]. Our findings are consistent with those of previous studies. Cytoplasmic RKIP expression has been found to positively correlate with survival in intestinal type gastric adenocarcinoma, but not in diffuse type [16].

The MAPK pathway, signal transducer and activator of transcription 3 (STAT3) pathway, and phosphatidylinositol 3-kinase (PI3K)/AKT/mammalian target of rapamycin (m-TOR) pathway are signaling pathways that regulate fundamental cellular processes such as proliferation, differentiation, angiogenesis, survival, apoptosis, and migration. Although each pathway is conceptually linear, considerable cross-talk occurs between the MAPK pathway and other signaling cascades [23]. MAPK signaling plays a central role in coordinating cell re-entry, cell survival and mortality, and cell invasion in response to growth factors. Expression of ERK is increased in gastric cancer tissue, and overexpression of ERK positively correlates with clinicopathological characteristics such as serosal invasion, lymph node involvement, and TNM stage [24]. In our study, overexpression of p-MEK and overexpression of p-ERK were observed in high proportions of tumours. Expression of p-ERK was slightly, but not significantly associated with survival, although p-MEK was not associated. The localization of p-ERK is an important factor in tumour progression, because activated ERK characteristically accumulates in the nucleus and transports extracellular stimuli from the cell surface to the nucleus in intracellular signal transducing pathways. MEK-catalysed ERK phosphorylation is necessary but not sufficient for the full nuclear localization response. Nuclear localization of phosphorylated ERK is affected by other proteins such as dual specificity phosphatase [25]. In colorectal cancer cells, the trafficking protein particle complex 4 (TRAPPC4) modulates the location of p-ERK to activate the relevant signaling pathway [26]. On the other hand, other studies reported that MAPK activity is rather suppressed in human gastric adenocarcinoma [27,28]. The complex multiple signaling MAPK pathway accepts many positive or negative stimuli, including negative auto-feedback mechanisms, and ERK activation is inhibited by components of the network, such as protein tyrosine phosphatase (PTP) or other MAPK phosphatases activated by transcription factors [29]. Consequently, ERK might not necessarily be activated when the direct upstream regulator MEK is active. Raf/MEK/ERK signaling pathway seems to be affected also by various regulators or negative feedback mechanisms. Therefore, the combined expression of upstream regulator and downstream effector may have an important impact on survival. In the present study, patients with negative RKIP expression had poorer survival (5-year RFS = 44%) than those with only positive RKIP expression (66%), patients with positive p-ERK expression had similar survival (49%) to those with negative p-ERK expression (75%), and patients with a combination of negative RKIP expression and positive p-ERK expression had poorer survival (33%) than those with positive RKIP expression or negative p-ERK expression (69%). In addition, negative RKIP and positive p-ERK expression was observed in 18 (69%) of 26 metastatic lymph nodes obtained from patients with recurrent disease. Our findings suggest that combined expression might be an independent prognostic factor.

ERK or MEK activation results from the sequential activation of a series of protein kinases, including Raf-1, and the up-regulating protein RAS. Approximately 30% of all human tumours have an activating mutation in a RAS gene. In particular, KRAS mutations are among the most common genetic abnormalities in several types of human cancer, including pancreatic cancer, colon cancer, and lung cancer [30]. In contrast, RAS mutations have been found in only a small proportion of human gastric cancers [31], implicating other mechanisms in the activation of RAS signaling in gastric tumourigenesis. B-RAF mutations are more narrowly distributed and are prevalent in a few specific malignancies, including melanoma, papillary thyroid cancer, and low-grade ovarian cancer, but are not found in gastric cancer [32,33]. In the present study, we focused on more downstream proteins such as MEK, ERK, and RAF inhibitors such as RKIP, and did not measure RAS or RAF expression. We previously showed that high expression of HER1 or HER3, which are upstream components of the RAS/RAF/MAPK and other tyrosine kinase pathways, was associated with poor survival in gastric cancer [34]. In addition, we reported that the expression of m-TOR in another pathway involving HER was related to survival in gastric cancer [35]. Signaling pathways involving tyrosine kinase receptors seem to be intimately related to invasion, metastasis, and outcomes in gastric cancer. However, anticancer agents that inhibit these pathways are not utilized clinically, with the exception of trastuzumab, an HER2 antagonist. Molecules implicated in downstream signaling pathways, such as ERK, may be targets for chemotherapy in advanced or metastatic gastric cancer. Small-molecule inhibitors of the MAPK cascade that are designed to target various steps of this pathway, such as MEK inhibitor and Raf inhibitor, have entered clinical trials, but direct ERK inhibitors have yet to be evaluated [36-39].

Many pathological and molecular assays suggest that gastric cancer is a heterogeneous disease. However, despite evidence indicating that gastric cancer is characterized by interindividual differences in tumour progression, histopathological features, and treatment response, a "one size fits all" approach to analysis has been used in many studies of gastric cancer, resulting in inconsistent outcomes [40]. The procurement of specimens from multiple sites may be essential when assessing heterogeneous tumours. We counted stained cancer cells in at least three fields per section, including the deepest site invaded by cancer cells, the surface of the lesion, and an intermediate zone. Staining for RKIP, p-MEK, or p-ERK often differed between the lesion surface and sites of deep invasive, or between differentiated and undifferentiated portions of the same lesion.

## Conclusions

In summary, loss of RKIP was associated with tumour progression and poor survival in gastric cancer. Furthermore, negative RKIP expression combined with positive p-ERK was an independent prognostic factor. Inhibition of the MAPK signaling pathway may thus become an important target for the treatment of gastric cancer.

## Competing interests

The authors declare that they have no competing interests.

## Authors' contributions

YF and MI designed experiments. YF, YK, and KK executed studies. YK and MI provided pathological analyses. YF wrote the manuscript which was edited by MI, KK, and KS. All authors read and approved the final manuscript. All authors read and approved the final manuscript.
